# Expression and ERG regulation of PIM kinases in prostate cancer

**DOI:** 10.1002/cam4.3893

**Published:** 2021-05-01

**Authors:** Sini K. Eerola, Annika Kohvakka, Teuvo L. J. Tammela, Päivi J. Koskinen, Leena Latonen, Tapio Visakorpi

**Affiliations:** ^1^ Faculty of Medicine and Health Technology Tampere University and Tays Cancer Center Tampere University Hospital Tampere Finland; ^2^ Department of Urology Tampere University Hospital Tampere Finland; ^3^ Department of Biology University of Turku Turku Finland; ^4^ Institute of Biomedicine University of Eastern Finland Kuopio Finland; ^5^ Fimlab Laboratories Ltd Tampere University Hospital Tampere Finland

**Keywords:** castration‐resistant prostate cancer, ERG, MYC, PIM kinases, prostate cancer

## Abstract

The three oncogenic PIM family kinases have been implicated in the development of prostate cancer (PCa). The aim of this study was to examine the mRNA and protein expression levels of PIM1, PIM2, and PIM3 in PCa and their associations with the *MYC* and *ERG* oncogenes. We utilized prostate tissue specimens of normal, benign prostatic hyperplasia (BPH), prostatic intraepithelial neoplasia (PIN), untreated PCa, and castration‐resistant prostate cancer (CRPC) for immunohistochemical (IHC) analysis. In addition, we analyzed data from publicly available mRNA expression and chromatin immunoprecipitation sequencing (ChIP‐Seq) datasets. Our data demonstrated that PIM expression levels are significantly elevated in PCa compared to benign samples. Strikingly, the expression of both PIM1 and PIM2 was further increased in CRPC compared to PCa. We also demonstrated a significant association between upregulated PIM family members and both the ERG and MYC oncoproteins. Interestingly, ERG directly binds to the regulatory regions of all *PIM* genes and upregulates their expression. Furthermore, ERG suppression with siRNA reduced the expression of PIM in PCa cells. These results provide evidence for cooperation of PIM and the MYC and ERG oncoproteins in PCa development and progression and may help to stratify suitable patients for PIM‐targeted therapies.

## Graphical abstract

Our data demonstrate for the first time that expression levels of all three PIM family kinases can be upregulated during prostate cancer progression and can thereby significantly contribute to this process, especially in cooperation with other co‐overexpressed oncoproteins, such as MYC and ERG, as shown here. The increased PIM expression levels may in turn be explained by our novel observation that ERG can induce transcription of all *PIM* family genes.

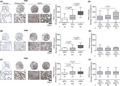

## 1 BACKGROUND

PIM kinases form a family of serine/threonine kinases consisting of three members, namely PIM1, PIM2, and PIM3, which have partially overlapping functions and expression patterns.^1^, ^2^, ^3^ PIM kinases are known to affect cancer progression by promoting proliferation, preventing apoptosis, and regulating the activities of several transcription factors. Increased expression of PIM family members has been detected both in hematopoietic malignancies and in solid tumors of epithelial origin, such as prostate cancer (PCa). PIM1 levels are elevated in PCa compared to benign prostatic epithelium,^4^, ^5^, ^6^, ^7^ with partially contrasting conclusions on whether PIM1 expression correlates with prostate tumor aggressiveness. Both PIM2 and PIM3 expression levels have been positively correlated with Gleason scores,^8^, ^9^, ^10^ although for PIM3, this has not yet been verified at the protein level. Furthermore, the expression levels of PIM kinases have not been determined in CRPC or characterized for all PIM family members in parallel in any prostate samples.

The *ERG* (ETS‐related gene 1) gene belongs to the ETS family of transcription factors and is fused with the prostate‐specific and androgen‐responsive *TMPRSS2* (transmembrane protease, serine 2) gene in approximately 50% of PCa cases, resulting in ERG overexpression.^11^ Additionally, two other *ERG* gene fusions can contribute to its increased expression, *SLC45A3*:*ERG* (solute carrier family 45, member 3) and *NDRG1*:*ERG* (N‐myc downstream regulated 1), which occur in less than 5% of PCa cases.^12^ Based on recent studies, *ERG* and *PIM1* are associated at the transcriptional level in PCa specimens. Moreover, ERG can directly bind to the *PIM1* promoter and thereby induce PIM1 expression.^13^

Overexpression of the *MYC* oncogene is one of the most common alterations in PCa.^14^, ^15^ PIM1 levels have been shown to be increased together with MYC levels during androgen ablation therapy.^16^ Furthermore, *PIM1* has been observed to enhance MYC‐induced tumorigenicity in human PCa in a mouse xenograft model,^17^ while coexpression of PIM1 and MYC in human PCa is associated with higher Gleason scores, suggesting that these oncoproteins synergize to induce advanced prostate carcinoma.^17^, ^18^ By contrast, there is no information available on the similar synergism of PIM2 or PIM3 with ERG or MYC oncoproteins.

The aim of this study was to systematically investigate in parallel how different PIM family members are expressed in primary and advanced PCa. In addition, we wanted to assess whether their expression levels are associated with those of the *MYC* or *ERG* oncogenes or with the prognosis of patients with PCa. We found that all PIM kinases are overexpressed in primary PCa and that PIM1 and PIM2 expression further increases in CRPC. Moreover, the expression of PIM kinases is regulated by ERG and associated with *MYC* expression.

## 2 MATERIALS AND METHODS

### 2.1 Patient samples

Altogether, 254 prostate tissue microarray (TMA) samples, including benign samples (*n* = 23) from adjacent tissue of untreated primary PCa prostatectomy samples, untreated primary PCas (*n* = 186), and locally recurrent CRPCs (*n* = 45), were obtained from Tampere University Hospital (TAUH, Tampere, Finland). The mean age of patients at diagnosis was 63.5 years (range: 49–72), and the mean prostate‐specific antigen (PSA) concentration was 14.3 ng/ml (range: 1.5–78.2) (Table S1). Biochemical progression was defined as two consecutive samples with PSA ≥0.5 ng/ml. The use of clinical material was approved by the Ethics Committee of the Tampere University Hospital and the National Authority for Medicolegal Affairs. For prospective sample collection, informed consent was obtained from all the subjects.

### 2.2 Gene correlation analyses

Two distinct clinical datasets were used to assess the gene expression levels of *PIM* genes and their associations with the *ERG* and *MYC* oncogenes in PCa patient samples: Tampere PCa RNA‐seq dataset^19^ and Integrative Genomic Profiling of Human Prostate Cancer microarray dataset.^20^

### 2.3 Immunohistochemical staining

PIM protein expression levels in prostate carcinomas were validated by immunohistochemical (IHC) analysis from formalin‐fixed paraffin‐embedded (FFPE) TMA samples. Primary antibodies against PIM1 (1:200, ab224772; Abcam), PIM2 (1:50, TA501166; OriGene Technologies Inc.), PIM3 (1:200, TA351349; OriGene), and ERG (1:200, EPR3864; Epitomics, Inc.) were used with the Histofine Simple Stain MAX PO multi; containing both Universal Immunoperoxidase Polymer Anti‐Mouse and Anti‐Rabbit (Nichirei Biosciences Inc.) secondary antibody according to the manufacturer's instructions. TMA sections were deparaffinized, and antigen retrieval was performed by autoclaving in TE buffer (5 mmol/L Tris‐HCl/ 1 mmol/L EDTA, pH 9) at 98°C for 15 min. The primary antibody was diluted in Antibody Diluent (ImmunoLogic). Staining was performed using a Lab Vision Autostainer 480S (Thermo Fisher Scientific). Sections were counterstained with Mayer's hematoxylin (Histolab AB) for 2 min and mounted with Neo‐Mount (Merck KGaA).

For negative controls, the primary antibody was omitted, and for positive controls, FFPE samples of tonsil, glioma, and/or colon tissues were used. Slides were scanned with an Olympus BX51 scanner with a 20× objective and Slide Strider software (Jilab Inc.) or with a NanoZoomer S60 Digital slide scanner (C13210‐01, Hamamatsu Photonics, K. K.) with a 20× objective. Nuclear scoring of the figures was performed with ImageJ® software (Wayne Rasband, NIH, USA) and its cell counter tool. Nuclear and cytoplasmic staining intensities of PIM proteins were classified on a scale from 0 to 3 with negative (0), weak (1), moderate (2), or strong (3) staining in proportion to the stained cancerous area. In the case of nuclear staining, if possible, a minimum of 200 cells were calculated from carcinogenic areas. The Histoscore (H‐score/HS) was calculated by a semiquantitative assessment of both the intensity of staining with the 0 to 3 scale and the percentage of positive PCa cells/area. The range of possible scores was from 0 to 300 or from 0 to 600 when both the cytoplasmic and nuclear scores were combined or summed. Samples stained against ERG antibody were categorized into ERG‐positive and ERG‐negative (Table S2 and Figure S1). The results from 85 ERG‐stained samples were already published in Leinonen et al. 2013,^21^ while 38 additional samples were stained and analyzed for these studies.

### 2.4 Cell culture

VCaP PCa cells (RRID:CVCL_2235) were kindly provided by Dr. Jack Schalken (Radboud University Nijmegen Medical Center). Cells were cultured as recommended by the suppliers and tested for mycoplasma contamination regularly.

### 2.5 Transfections for gene knockdown

siRNAs targeting *ERG* (sense: UGAUGUUGAUAAAGCCUAUU; antisense: UAGGCUUUAUCAACAUCAUU) or a negative control siRNA (MISSION siRNA Universal Negative Control #2) were purchased from Sigma‐Aldrich. The transfection reagent Lipofectamine RNAiMAX (Invitrogen) was used for transfecting siRNAs according to the manufacturer's instructions. VCaP PCa cells were reverse‐transfected with 25 nM siRNA and grown for 72 h before RNA and protein extraction.

### 2.6 Quantitative reverse transcription PCR (qRT‐PCR)

For determination of *ERG* and *PIM* mRNA expression levels, total RNA was extracted using TRIzol reagent (Invitrogen, Carlsbad, CA, USA) according to the manufacturer's protocol. qRT‐PCR was performed using random hexamer primers (Thermo Fisher Scientific, Waltham, MA, USA), Maxima reverse transcriptase (Thermo Fisher Scientific), Maxima SYBR Green qPCR Master Mix (Thermo Fisher Scientific), and the CFX96™ Real‐Time PCR Detection System (Bio‐Rad Laboratories, Inc.). The expression levels were measured from three biological and technical replicates and normalized against mRNA of the TATA‐binding protein (*TBP*). All primers are presented in Table S3.

### 2.7 Western blot analysis

After knockdown experiments, cells were lysed in Triton‐X lysis buffer containing 50 mM Tris‐HCl, pH 7.5, 150 mM NaCl, 0.5% Triton X‐100, 1 mM PMSF, 1 mM DTT, and 1× Halt protease inhibitor cocktail (Thermo Fisher Scientific), after which the lysates were sonicated four times for 30 s at medium power with Bioruptor equipment (Diagenode Inc.), and cellular debris was removed by centrifugation. Samples were resuspended in 2× Laemmli sample buffer and heated at 95°C for 5 min. Proteins were separated by Mini‐PROTEAN TGX Precast Gels (Bio‐Rad), and immobilized onto PVDF membranes (Immobilon‐P, Millipore, Merck). Primary antibodies against PIM1 (1:2000, Abcam, ab224772), PIM2 (1:2000, OriGene, TA501166), PIM3 (1:1000, OriGene, TA351349), ERG (1:5000, EPR3864; Epitomics), β Tubulin (1:40 000, Sigma‐Aldrich), or Fibrillarin (1:1000, Cell Signaling Technology) were used together with anti‐mouse HRP‐conjugated antibody produced in rabbit (1:10 000; DAKO) or anti‐rabbit HRP‐conjugated antibody produced in swine (1:5000; DAKO). Chemiluminescence reactions were generated using either Amersham^TM^ ECL Plus or ECL Prime reagents (GE Healthcare Life Sciences).

### 2.8 Statistical analyses

Statistical analyses for IHC protein expression levels were performed using the Mann‐Whitney U test. Gleason scores were divided into three groups: low (scores <7), intermediate (scores equal to 7), and high (scores >7 [from 8 to 10]). Correlations between PIM1/PIM2 or PIM3 expression and MYC were tested using Pearson's correlation coefficient. Grubbs’ test, also called the extreme studentized deviate (ESD) method, was used to analyze possible outliers from the *PIM*‐*MYC* gene correlation dataset, and a *p*‐value of 0.05 was used as a cutoff for the significance of the outliers. Associations between PIM1/PIM2 or PIM3 expression and ERG were tested with the Chi‐square test or Fisher's exact test depending on the form of data suitable for each analysis. Kaplan–Meier survival analysis and the log‐rank (Mantel‐Cox) test were used to estimate the progression‐free (PSA‐free) time (survival) between samples divided by their median expression into PIM low and PIM high expression groups. Unpaired two‐tailed Student's *t*‐test was used to calculate the significance between the control and experimental conditions in qRT‐PCR. All statistical analyses were performed using GraphPad Prism version 5.02 (GraphPad Software Inc). *p*‐values <0.05 (*), *p*‐values <0.01 (**), and *p*‐values <0.001 (***) were considered statistically significant.

To investigate the binding sites of ERG in all *PIM* promoter areas, we used a publicly available dataset (GSM353647^22^) with Integrative Genomics Viewer (IGV) version 2.5.0 (Broad Institute) to observe ERG ChIP‐seq peaks compared to *PIM* regulatory regions in VCaP PCa cells.

## 3 RESULTS

### 3.1 *PIM* gene expression is elevated in prostate cancer

**FIGURE 1:**
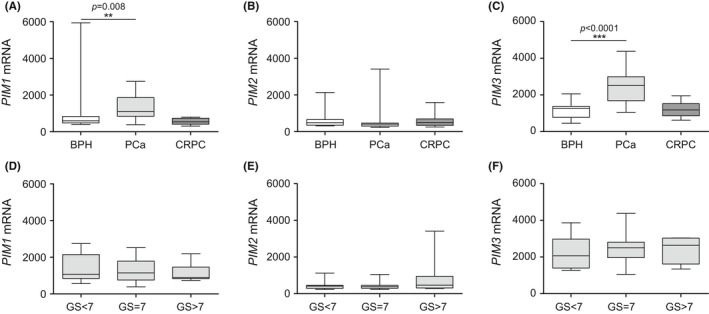
Expression of *PIM1* and *PIM3* is elevated in primary PCa. The Tampere PCa sequencing dataset^19^ was used to assess the mRNA expression levels of the *PIM1* (A, D), *PIM2* (B, E), and *PIM3* (C, F) genes. The results were first categorized into BPH (*n* = 12), primary PCa (*n* = 30), and CRPC (*n* = 13) samples (A–C). Primary PCa samples were further divided based on Gleason scores GS<7 (*n* = 7), GS = 7 (*n* = 7), and GS>7 (*n* = 15) (D–F). Error bars display the minimum and maximum values, and the line inside the boxes displays the median in the dataset range. *p*‐values <0.05 (*), *p*‐values <0.01 (**), and *p*‐values <0.001 (***) were considered statistically significant

To study the expression of all *PIM* family members in PCa, we first utilized our RNA‐seq‐based mRNA expression dataset of PCa patient samples (Tampere PCa sequencing data^19^). Of all the *PIM* members, the overall expression of *PIM3* was the highest, and *PIM2* was the lowest (Figure S2A). Similar results were observed in another dataset ^20^ (Figure S2B). Next, we analyzed transcriptional expression levels according to pathology (BPH, PCa, and CRPC). In our Tampere PCa dataset, there was a significant increase in *PIM1* and *PIM3* but not *PIM2* gene expression in PCa compared to BPH patient samples (Figure 1A–C). When the primary tumors were categorized according to Gleason scores (GS < 7, GS = 7, and GS > 7), a slight but not statistically significant increase was detected for *PIM2* in samples with Gleason scores higher than 7 when compared to samples with lower Gleason scores (Figure 1E), while no association with Gleason scores was observed for *PIM1* or *PIM3* expression levels (Figure 1D and F). We analyzed also larger Taylor et al. microarray dataset and the results were parallel with our own cohort but not statistically significant (Figure S3).

### 3.2 PIM protein expression increases during the prostate cancer progression

**FIGURE 2:**
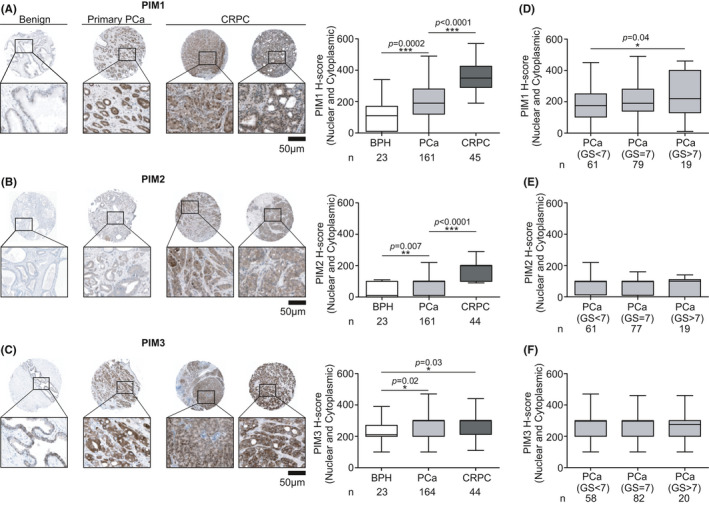
PIM protein levels are upregulated during PCa progression. IHC staining was performed for FFPE TMA samples of 23 benign prostate, 186 primary PCa, and 45 CRPC samples. Representative IHC figures of whole TMA spots with 5x and 20x enlargement of the refined area are shown from benign prostate, primary PCa and CRPC samples stained with PIM1 (A), PIM2 (B), and PIM3 (C) antibodies. Boxplots were made from IHC staining results by combined Histoscore numbers of nuclear and cytoplasmic staining of the samples. Primary PCa samples were categorized by Gleason scores (GS<7, GS = 7, and GS>7) and PIM1 (D), PIM2 (E), and PIM3 (F) protein expression levels. Error bars display the minimum and maximum values, and the line inside the boxes displays the median in the dataset range. Sample numbers (*n*) and *p*‐values (*p*) are marked in the figures. *p*‐values <0.05 (*), *p*‐values <0.01 (**), and *p*‐values <0.001 (***) were considered statistically significant

Next, we wanted to assess PIM expression levels at the protein level using a sample cohort containing 23 benign adjacent tissue samples from the primary PCa samples, 186 primary PCa samples, and 45 CRPC samples. Our results from IHC analysis showed a significant increase in PIM1 and PIM2 protein expression levels in primary PCa compared to benign patient samples (*p *= 0.0002, *p *= 0.007; Figure 2A and B). However, the expression levels of either PIM1 or PIM2 had no association with progression‐free survival (*p *= 0.77, *p *= 0.07; Figure S4A and B). To our knowledge, PIM3 protein expression levels in PCa have not been reported before. Our results show that the PIM3 levels were significantly higher in PCa than in benign samples (*p *= 0.02; Figure 2C). Additionally, in this case, the expression levels did not correlate with progression‐free survival (*p *= 0.8; Figure S4C). When primary PCa samples of different Gleason score groups were compared, a statistically significant increase was observed in PIM1 expression with Gleason scores higher than 7 when compared to Gleason scores lower than 7 (*p *= 0.04; Figure 2D). However, no statistically significant differences in the PIM2 and PIM3 protein expression levels were observed within the different Gleason score groups (Figure 2E and F).

In CRPC samples, both PIM1 and PIM2 expression levels were significantly upregulated compared to those in primary PCa patient samples (*p*<0.0001, *p*<0.0001; Figure 2A and B). The PIM3 expression level was significantly higher in CRPC than in BPH (*p *= 0.03), while no further increase was observed from primary PCa to CRPC (Figure 2C). Thus, our data indicate that the expression of PIM1 and PIM2 increases during the progression of the disease.

### 3.3 Expression of *PIM1 or PIM3* and *MYC* oncogene positively correlate in prostate cancer

**FIGURE 3:**
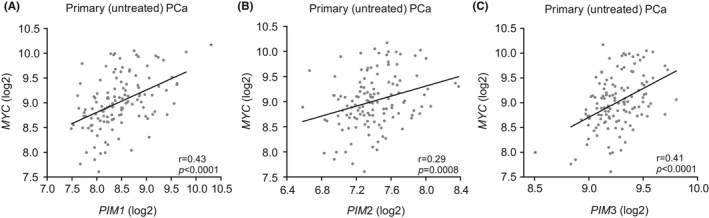
*PIM* and *MYC* oncogene expression is associated with human PCa. The Integrative Genomic Profiling of Human Prostate Cancer microarray dataset^20^ (*n* = 126) was used to assess the mRNA expression of the *PIM1* (A), *PIM2* (B), and *PIM3* (C) genes and their correlations with *MYC* oncogene in logarithmic scale in untreated prostate cancer patient samples. Possible outliers of the dataset were calculated with Grubbs’ test and marked as a black star in the dot blot. *p*‐values (*p*) and Pearson correlation values (r) are marked in the figures. *p*‐values <0.05 (*), *p*‐values <0.01 (**), and *p*‐values <0.001 (***) were considered statistically significant

As PIM1 kinase has been shown to cooperate with the MYC oncoprotein to induce advanced PCa,^17^ we wanted to investigate the possible associations between the expression of distinct *PIM* family genes and the *MYC* oncogene. We observed correlations between *PIM1* (*r* = 0.43; Figure 3A), *PIM2* (*r* = 0.29, Figure 3B), and *PIM3* (*r* = 0.41; Figure 3C) with *MYC* mRNA in the Taylor et al. 2010 dataset. This correlation was confirmed in our smaller Tampere PCa cohort for *PIM3* but not for *PIM1* or *PIM2* (Figure S5A–C). These results suggest for the first time that not only PIM1, but also PIM3 may cooperate with MYC in prostate tumorigenesis.

### 3.4 Expression of PIM genes and proteins is associated with ERG

**FIGURE 4:**
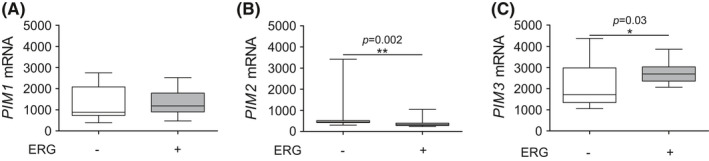
Association of the *PIM* and *ERG* oncogenes. Using the Tampere PCa cohort,^19^ *PIM1* (A), *PIM2* (B), and *PIM3* (C) mRNA expression levels were compared in *ERG*‐negative (*n* = 15) and *ERG*‐positive (*n* = 15) samples. The cutoff for *ERG*‐negative and *ERG*‐positive expression values was calculated from the *ERG* expression average of BPH samples by adding double the standard deviation to this value. Error bars display the minimum and maximum values, and the line inside the boxes displays the median in the dataset range

Next, we assessed *PIM* associations with *ERG* at the transcriptional level in primary tumors. No significant association at the transcriptional level was detected between *PIM1* and *ERG* in the Tampere PCa dataset (Figure 4A), while the association between *PIM2* and *ERG* was significantly negative (Figure 4B). Interestingly, *PIM3* and *ERG* showed a significant positive association (Figure 4C). In contrast, in the larger Taylor et al. dataset, *ERG* showed a significant association with *PIM1* but not with *PIM2* or *PIM3* gene expression in primary untreated PCa samples (Figure S6A–C). Taken together, these results suggest a cooperative or regulatory role between the *PIM* and *ERG* oncogenes.

**FIGURE 5:**
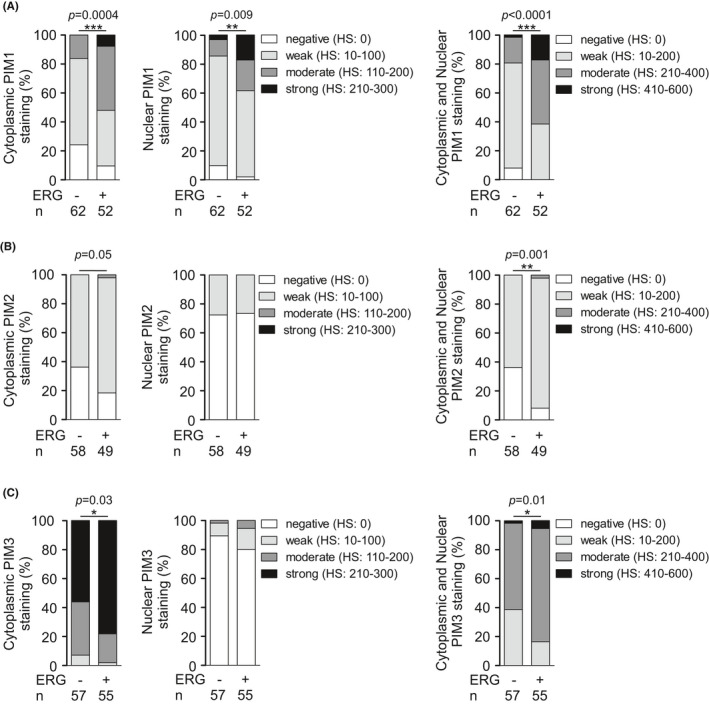
PIM kinases are associated with the ERG oncoprotein. Histograms of PIM1 (A), PIM2 (B), and PIM3 (C) protein expression levels in the cytoplasmic, nuclear, or both compartments were categorized into negative, low, moderate, and strong staining intensities and compared between ERG‐negative and ERG‐positive samples

Next, we wanted to investigate the possible associations of PIM and ERG at the protein level. Based on IHC staining, all PIM family members showed an association with ERG in PCa patient specimens. Higher nuclear, cytoplasmic, or both nuclear and cytoplasmic PIM1 expression was associated with ERG positivity (*p *= 0.0004, *p *= 0.0009, *p*<0.0001, Figure 5A). Moreover, significantly higher combined cytoplasmic and nuclear PIM2 expression were associated with the expression of ERG (*p *= 0.001; Figure 5B), and higher cytoplasmic and combined cytoplasmic and nuclear PIM3 expression were significantly associated with ERG expression (*p *= 0.03, *p *= 0.01; Figure 5C), while for PIM2 and PIM3, an association was not observed in samples with only nuclear staining (Figure 5B, C). Altogether, these results at both the mRNA and protein levels indicate that in addition to PIM1, PIM2 and PIM3 are also associated with the expression of the ERG oncogene.

### 3.5 Expression of all *PIM* family members is regulated by ERG

**FIGURE 6:**
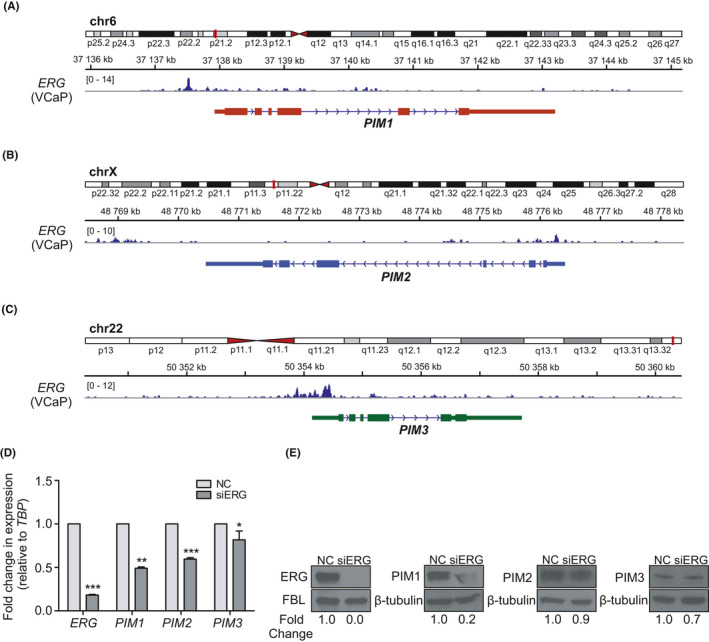
ERG binds to the regulatory regions of all PIM genes and regulates their expression. Publicly available ChIP‐seq data were used to determine the binding sites for ERG on the *PIM1* (A), *PIM2* (B), and *PIM3* (C) promoter areas. D. qPCR was performed on ERG‐silenced (siERG; 25 nM) VCaP cells from which *ERG*, *PIM1*, *PIM2*, and *PIM3* transcriptional expression levels were determined after 72 h and compared with cells transfected with control siRNA (NC). *TBP* was used as a reference gene to normalize the data. E. Western blot analyses of *ERG*‐silenced (siERG; 25 nM) VCaP cells, from which ERG, PIM1, PIM2, and PIM3 protein expression levels were determined after 72 h and compared with cells transfected with control siRNA (NC). Fold changes in protein expression levels were normalized against fibrillarin (FBL) or β‐tubulin, which were used as loading controls

The strong associations between ERG and PIM kinases led us to further investigate the nature of the cooperation between them. Previous data by Magistroni et al. 2011 demonstrated direct binding of the TMRSS2:ERG fusion protein to the *PIM1* promoter, enabling ERG‐mediated regulation of *PIM1* expression in benign RWPE‐1 prostate cells. Therefore, we used a publicly available ERG ChIP‐seq dataset from VCaP PCa cells^22^ to assess the possible ERG binding sites at the *PIM1*, *PIM2*, and *PIM3* loci. This analysis revealed multiple ERG binding sites not only at *PIM1* but also at the *PIM2* and *PIM3* promoter regions (Figure 6A–C).

To assess the effect of ERG on the transcriptional regulation of *PIM* genes, we performed qRT‐PCR of VCaP cells transfected with *ERG* siRNA (siERG) or scrambled negative control (NC) siRNA. The results showed significant transcriptional downregulation of all *PIM* mRNAs in *ERG*‐silenced samples compared to control samples (Figure 6D). This downregulation was also evident at the protein level in immunoblotted samples (Figure 6E). Altogether, these results indicate that ERG regulates the expression of not only PIM1 but also PIM2 and PIM3.

## 4 DISCUSSION

To be able to improve PCa therapies, it is important to identify the critical oncogenes that promote cancer development toward a more aggressive and possibly lethal form. This in turn may help to recognize high‐risk CRPC patients from localized PCa at an earlier stage and thereby choose the right types of therapies to increase patient survival. To achieve this goal, new molecular biomarkers and drug targets are needed.

This study provides novel insights into the role of different PIM family kinases together with other effective oncoproteins involved in PCa progression. Here, we have for the first time compared the mRNA and protein expression of all PIM family members in PCa patient samples in parallel. This is important, as expression analyses focusing on only one of the functionally fairly redundant family members may underestimate the overall contribution of PIM kinases to PCa progression. At the transcriptional level, there was a slight increase in *PIM1* and a more robust upregulation of *PIM3* mRNAs in primary PCa patient samples compared to normal or BPH samples in our PCa cohort.^19^ At the protein level, however, the expression levels of all PIM kinase family members are elevated in primary PCa compared to benign prostate samples and are further increased in CRPC samples for both PIM1 and PIM2. PIM1 protein levels also increased in a Gleason score‐dependent manner. To our knowledge, PIM3 protein levels have not been analyzed in PCa before, nor have the levels of any PIM family proteins in CRPC. However, there was no association between any PIM expression and progression‐free survival in our dataset.

In addition to our Tampere PCa RNA‐seq data, we utilized Integrative Genomic Profiling of Human Prostate Cancer microarray data.^20^ When overall *PIM* mRNA expression levels were compared with this larger dataset, the results were fairly similar to our Tampere PCa dataset, although no significant differences were detected between the samples from normal prostates and primary prostate tumors or metastasized CRPC tumors, and no Gleason score‐dependent differences were detected. These discrepancies may partly be due to differences between the platforms used (RNA‐seq vs. microarray) or in the samples assessed (BPH vs. normal prostate tissue and CRPC vs. metastasized CRPC). Further clinical datasets will undoubtedly shed more light on the matter.

Based on earlier results, both PIM1 and MYC levels are elevated in human PCas,^4^, ^5^, ^6^, ^7^, ^14^, ^15^ suggesting that they may cooperate in prostate carcinogenesis. Moreover, it has been discovered that PIM1 can enhance the transcriptional activity of MYC and thereby promote tumorigenicity.^17^ Aligned with the previously published data, we observed positive correlation of *PIM1* and *MYC* expression within Taylor et al. dataset. However, in our own dataset, the correlation between *PIM1* and *MYC* was not statistically significant. *PIM2* and *MYC* showed only weak positive correlation in Taylor et al. cohort and no significant correlation was detected in our own dataset. The discrepancies between the two datasets in case of *PIM1*/*PIM2* and *MYC* may partly be due to the different size of the cohorts (Taylor et al. *n* = 126 and our cohort *n* = 30) and differences between the platforms used (RNA‐seq vs. microarray) or in the samples assessed. Further clinical validation will undoubtedly shed more light on the matter. However, in this study, we show a positive correlation between the expression levels of *PIM3* and *MYC* mRNAs within the two human PCa datasets, suggesting that PIM3 and MYC also cooperate in PCa progression. While MYC is a challenging target for therapies, patients overexpressing both PIM and MYC proteins may benefit from PIM‐targeted therapy.

In addition to the *MYC* oncogene, it is known that the transcription factor *ERG* is often coexpressed with PIM1 and that ERG binds to the *PIM1* promoter and directly induces its expression.^13^ Here we also show a significant association between *ERG* and *PIM3* gene expression in our PCa RNA‐seq dataset and demonstrate that all PIM kinases are associated to a significant extent with ERG at the protein level. Furthermore, we show that there are ERG binding sites on the regulatory regions of all the *PIM* family members and that ERG regulates their expression levels, as confirmed by reduced PIM mRNA and protein levels by RNA interference‐mediated *ERG* knockdown. This regulation in turn may be relevant for ERG‐induced prostate tumorigenesis.

In a novel publication by Luszczak and others,^23^ it was reported that both the PIM and PI3 K/AKT/mTOR pathways are overlapping and cross‐impact each other. Luszczak and others^23^ also suggested that more effort should be put into identifying the associating oncogenes/biomarkers of each patient and targeted combinatorial treatments against them. Indeed, there are already promising results from combinatorial treatment against PIM and PI3 K in PIM‐upregulated and TMPRSS:ERG‐fusion‐positive PCa cells.^24^ Based on our findings, these combinatorial treatments against PIM, ERG, and MYC signaling pathways in relevant patients may be helpful.

## 5 CONCLUSIONS

In this study, we demonstrate for the first time that the mRNA and protein expression levels of all three PIM family kinases can be upregulated during PCa progression and can thereby significantly contribute to this process, especially in cooperation with other co‐overexpressed oncoproteins, such as MYC and ERG, as shown here. The increased PIM expression levels may in turn be explained by our observation that ERG can induce transcription of all PIM family genes. As ERG itself is often overexpressed in PCa due to oncogenic gene fusions, our data suggest that it is important to identify patients who express high levels of any PIM kinase together with other oncoproteins, such as MYC or ERG, as those patients may benefit most from targeted and combinatorial therapies.

## CONFLICT OF INTEREST

The authors declare that they have no conflict of interest.

## ETHICS APPROVAL AND CONSENT TO PARTICIPATE

The use of clinical material was approved by the ethics committee of the Tampere University Hospital (Tampere, Finland) and the National Authority for Medicolegal Affairs. For prospective sample collection, informed consent was obtained from the subjects.

## Supporting information

Fig S1

Fig S2

Fig S3

Fig S4

Fig S5

Fig S6

Table S1

Table S2

Table S3

## Data Availability

The Integrative Genomic Profiling of Human Prostate Cancer microarray dataset^20^ is available at https://www.ncbi.nlm.nih.gov/geo/query/acc.cgi?acc=GSE21036 under the accession number GSE21036. To investigate the binding sites of ERG oncogene in all PIM kinase promoter areas, we used a publicly available dataset https://www.ncbi.nlm.nih.gov/geo/query/acc.cgi?acc=GSE14092 under the accession number GSM353647^22^; Tampere PCa RNA‐seq data^19^ and datasets for IHC analysis are mainly available in the Supplementary Information, and additional information related to these datasets are available from the corresponding author upon reasonable request.
