# Supplementary figures and images for: Expression and ERG regulation of PIM kinases in prostate cancer
Source: Cancer Med. 2021 May 1;10(10):3427–36. doi: 10.1002/cam4.3893 (PMC8124112; doi:10.1002/cam4.3893)

# Supplementary Figure S1.

Primary PCa

ERG

+

-

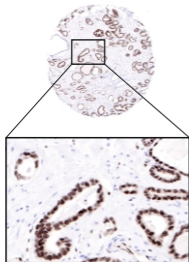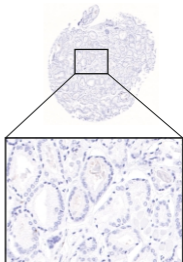

50  $\mu$ m

Supplement: Supplementary file 1 — Fig S1 [file CAM4-10-3427-s002.pdf]

# Supplementary Figure S2

**A**

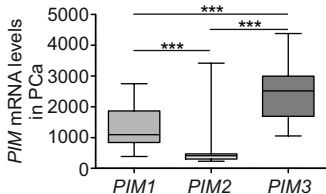

**B**

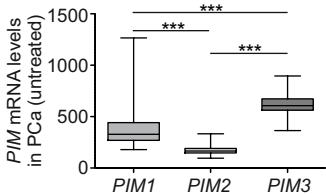

Supplement: Supplementary file 2 — Fig S2 [file CAM4-10-3427-s007.pdf]

**Supplementary Figure S3**

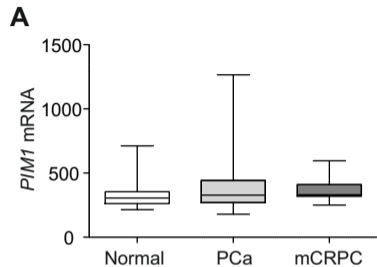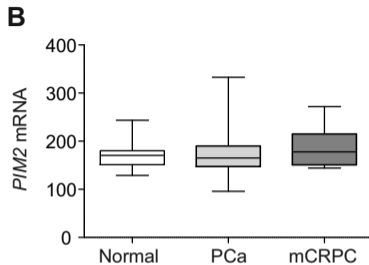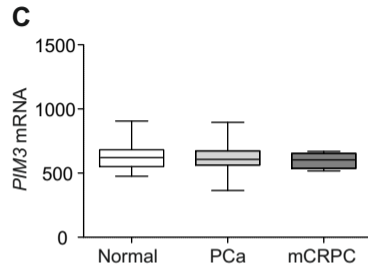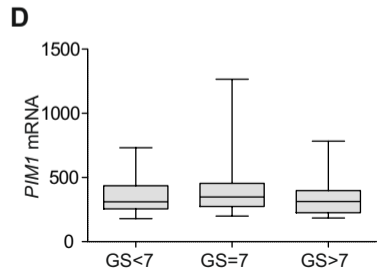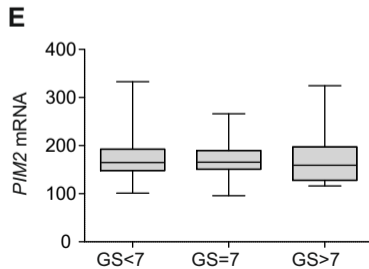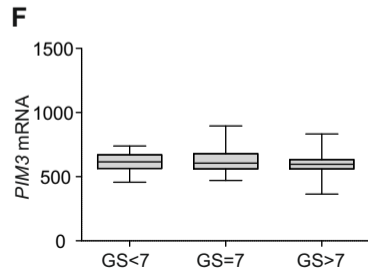

Supplement: Supplementary file 3 — Fig S3 [file CAM4-10-3427-s008.pdf]

# Supplementary Figure S4

**A**

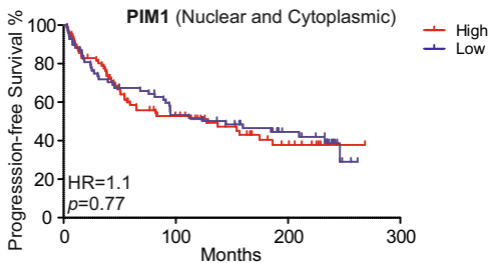

**B**

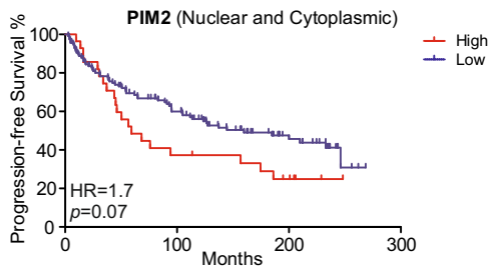

**C**

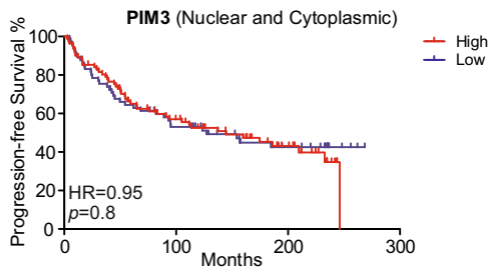

Supplement: Supplementary file 4 — Fig S4 [file CAM4-10-3427-s004.pdf]

# Supplementary Figure S5

**A**

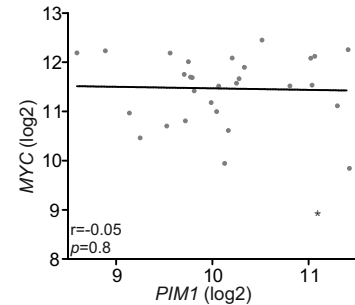

**B**

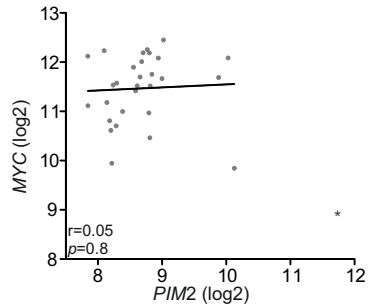

**C**

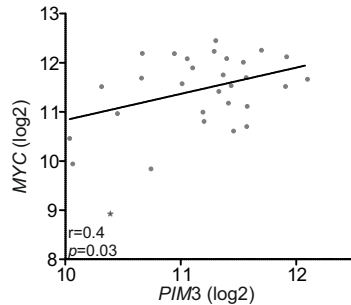

Supplement: Supplementary file 5 — Fig S5 [file CAM4-10-3427-s003.pdf]

# Supplementary Figure S6

**A**

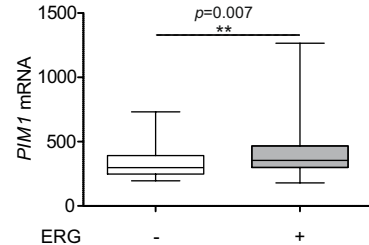

**B**

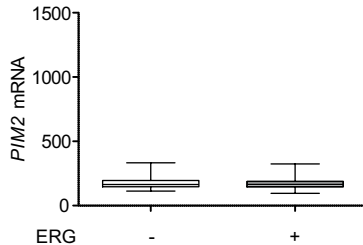

**C**

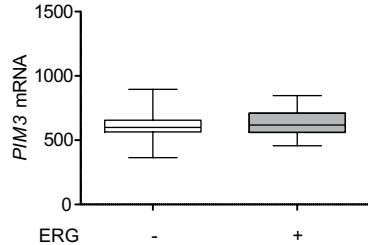

Supplement: Supplementary file 6 — Fig S6 [file CAM4-10-3427-s006.pdf]
