# Supplementary material for: Expression and ERG regulation of PIM kinases in prostate cancer
Source: Cancer Med. 2021 May 1;10(10):3427–36. doi: 10.1002/cam4.3893 (PMC8124112; doi:10.1002/cam4.3893)
Supplement: Supplementary file 7 — Table S1 [file CAM4-10-3427-s001.docx]

**Supplementary Table S1. PCa patient data**

The number and clinicopathological description of the primary PCa prostatectomy samples used in the study.

| **PCa prostatectomy specimens, n** **186**  Gleason score, n (%)  <7 67 (36)  7 95 (51)  >7 22 (12)  pT stage, n (%)  pT1 1 (0.5)  pT2 115 (62)  pT3 68 (37)  pT4 1 (0.5)  Mean age at diagnosis 63.5 years (median 64.0, range 49.0–72.0 years)  Mean PSA at diagnosis 14.3 ng/mL (median 10.5, range: 1.5–78.2 ng/mL)  Median follow-up time 94.9 months (range 2.8–268.6 months) |
| --- |
