# Supplementary material for: Expression and ERG regulation of PIM kinases in prostate cancer
Source: Cancer Med. 2021 May 1;10(10):3427–36. doi: 10.1002/cam4.3893 (PMC8124112; doi:10.1002/cam4.3893)
Supplement: Supplementary file 8 — Table S2 [file CAM4-10-3427-s009.docx]

**Supplementary Table S2. IHC data**

Number of the immunohistochemical staining description of the prostatectomy samples. † HS, Histo-Score.

| **PIM1**  Adjacent benign prostate sample, n = 23  Cytoplasmic HS Nuclear HS both Cytoplasmic and Nuclear HS  Range: 0–200 0–240 0–340  Median: 70 10 110  Mean: 56 10 105  Primary PCa samples, n = 161  Cytoplasmic HS Nuclear HS both Cytoplasmic and Nuclear HS  Range: 0–300 0–300 0–490  Median: 100 70 190  Mean: 114 88 202  CRPC samples, n = 45  Cytoplasmic HS Nuclear HS both Cytoplasmic and Nuclear HS  Range: 100–300 0–300 190–570  Median: 200 200 350  Mean: 178 183 361 |
| --- |
| **PIM2**  Adjacent benign prostate sample, n = 23  Cytoplasmic HS Nuclear HS both Cytoplasmic and Nuclear HS  Range: 0–100 0–10 0–110  Median: 70 10 110  Mean: 40 0 45  Primary PCa samples, n = 161  Cytoplasmic HS Nuclear HS both Cytoplasmic and Nuclear HS  Range: 0–200 0–60 0–220  Median: 100 0 100  Mean: 69 5 74  CRPC samples, n = 44  Cytoplasmic HS Nuclear HS both Cytoplasmic and Nuclear HS  Range: 90–210 0–90 90–290  Median: 200 0 200  Mean: 160 3 164 |
| **PIM3**  Adjacent benign prostate sample, n = 23  Cytoplasmic HS Nuclear HS both Cytoplasmic and Nuclear HS  Range: 100–200 0–120 100–390  Median: 200 10 210  Mean: 190 39 228  Primary PCa samples, n = 164  Cytoplasmic HS Nuclear HS both Cytoplasmic and Nuclear HS  Range: 100–300 0–210 100–470  Median: 300 0 300  Mean: 254 13 267  CRPC samples, n = 44  Cytoplasmic HS Nuclear HS both Cytoplasmic and Nuclear HS  Range: 100–300 0–240 110–440  Median: 300 0 300  Mean: 251 11 262 |
| **ERG**  Primary PCa samples, n 123  ERG negative ERG positive  n: 67 56 |
