# Supplementary material for: Expression and ERG regulation of PIM kinases in prostate cancer
Source: Cancer Med. 2021 May 1;10(10):3427–36. doi: 10.1002/cam4.3893 (PMC8124112; doi:10.1002/cam4.3893)
Supplement: Supplementary file 9 — Table S3 [file CAM4-10-3427-s005.docx]

**Supplementary Table S3. Primers for qRT-PCR.**

Sequences of all the primers used in the study.

***PIM1* primers for qRT-PCR**

| F: 5’ CTGGGGAGAGCTGCCTAATG 3’ |
| --- |
| R: 5’ GCTCCCCTTTCCGTGATGAA 3’ |

***PIM2* primers for qRT-PCR**

| F: 5’ TGACTTTGATGGGACAAGGGT 3’ |
| --- |
| R: 5’ GGAATGTCCCCACACACCAT 3’ |

***PIM3* primers for qRT-PCR**

| F: 5’ ACCGACTTCGACGGCAC 3’ |
| --- |
| R: 5’ TATCGTAGAGAAGCACGCCC 3’ |

***TPB* primers for qRT-PCR**

| F: 5’ GAATATAATCCCAAGCGGT 3’ |
| --- |
| R: 5’ ACTTCACATCACAGCTCCCC 3’ |
